# Should we remain hopeful? The key 8 weeks: spatiotemporal epidemic characteristics of COVID-19 in Sichuan Province and its comparative analysis with other provinces in China and global epidemic trends

**DOI:** 10.1186/s12879-020-05494-6

**Published:** 2020-11-05

**Authors:** Xinyin Xu, Jing Zeng, Runyou Liu, Yang Liu, Xiaobo Zhou, Lijun Zhou, Ting Dong, Yuxin Cha, Zhuo Wang, Ying Deng, Yu Zhang, Liao Feng, Chen Pu, Xianping Wu, Bo Zhong

**Affiliations:** 1 Sichuan Center for Disease Control and Prevention, No. 6 Zhongxue Rd, Chengdu, 610041 China; 2 University of Texas Health Science Center at Houston, Houston, USA

**Keywords:** COVID-19, SARS-CoV-2, Sichuan Province, China, Global, Temporal and spatial distribution, Epidemiological characteristics, Spatial autocorrelation

## Abstract

**Background:**

The COVID-19 spread worldwide quickly. Exploring the epidemiological characteristics could provide a basis for responding to imported cases abroad and to formulate prevention and control strategies in areas where COVID-19 is still spreading rapidly.

**Methods:**

The number of confirmed cases, daily growth, incidence and length of time from the first reported case to the end of the local cases (i.e., non-overseas imported cases) were compared by spatial (geographical) and temporal classification and visualization of the development and changes of the epidemic situation by layers through maps.

**Results:**

In the first wave, a total of 539 cases were reported in Sichuan, with an incidence rate of 0.6462/100,000. The closer to Hubei the population centres were, the more pronounced the epidemic was. The peak in Sichuan Province occurred in the second week. Eight weeks after the Wuhan lockdown, the health crisis had eased. The longest epidemic length at the city level in China (except Wuhan, Taiwan, and Hong Kong) was 53 days, with a median of 23 days. Spatial autocorrelation analysis of China showed positive spatial correlation (Moran’s Index > 0, *p* < 0.05). Most countries outside China began to experience a rapid rise in infection rates 4 weeks after their first case. Some European countries experienced that rise earlier than the USA. The pandemic in Germany, Spain, Italy, and China took 28, 29, 34, and 18 days, respectively, to reach the peak of daily infections, after their daily increase of up to 20 cases. During this time, countries in the African region and Southeast Asian region were at an early stage of infections, those in the Eastern Mediterranean region and region of the Americas were in a rapid growth phase.

**Conclusions:**

After the closure of the outbreak city, appropriate isolation and control measures in the next 8 weeks were key to control the outbreak, which reduced the peak value and length of the outbreak. Some countries with improved epidemic situations need to develop a continuous “local strategy at entry checkpoints” to to fend off imported COVID-19.

## Background

Faced with unknown infectious diseases, policymakers are always looking for the best point of prevention and control, balancing between underestimating and overestimating the risk. In December 2019, there were reports of unexplained pneumonia infections in Wuhan, China [1]. On December 31, experts from the Chinese Center for Disease Control and Prevention (CDC) went to Wuhan to learn about the situation and collected samples from patients [2]. On January 3, 2020, scientists from the National Institute for Viral Disease Control and Prevention identified the sequence of novel β-genus coronaviruses from specimens taken from patients in Wuhan and later published this information to the public. The study found that the patients were infected with β-genus coronaviruses with genetic characteristics different from SARS-CoV and MERS-CoV that was temporarily named the 2019 novel coronavirus (2019-nCoV) and later renamed severe acute respiratory syndrome coronavirus 2 (SARS-CoV-2). The disease caused by it is called coronavirus disease (COVID-19) [3, 4]. Subsequently, cases of COVID-19 were reported in 31 provinces of China, Hong Kong, Macao, Taiwan and other countries. Facing the grim situation of the global COVID-19 outbreaks, this study focuses on analysing the overall epidemic characteristics of Sichuan Province, which is close to Wuhan in the east. This study also analysed the overall epidemic characteristics of China, selected key dates for comparison, considered the epidemic laws, and analysed the global epidemic trends to provide a basis for China to cope with imported cases abroad and for other countries to grasp the timing of prevention and control to formulate appropriate strategies.

## Methods

To reflect the actual geographical distribution risk of confirmed cases in Sichuan Province, the current residential addresses of confirmed cases from the Sichuan COVID-19 Surveillance System were selected for distribution analysis. For comparison with neighbouring provinces, the situation of confirmed cases reported by the National Health Commission [5], Hubei Province [6], and the health commissions of other provinces were analysed using the address of the reporting unit (usually the hospital or CDC). Data from various countries were taken from the WHO COVID-19 Daily Report published since January 21 [7]. The number of cases in China before that first daily report and the first case information in Thailand, Japan, the Republic of Korea, and the USA, mentioned in the previous report of WHO, was taken from their national government websites [8–11]. The number of counties and districts of the cities in Sichuan Province were taken from the Statistical Yearbook of Sichuan Province [12]. The population of various cities in China were taken from the Statistical Yearbook of kinds of provinces.

The characteristics of cumulative confirmed cases, daily new cases, morbidity, etc. were compared based on temporal and spatial factors; the length of time from the first case report to the end of the local cases (i.e., nonforeign imported cases), abbreviated hereafter as “epidemic length”, was analysed to explore the influencing factors of prevention and control of the outbreaks. Key analysis times and units were set based on key dates of policy and disease incubation periods. The epidemic maps of Sichuan and China were drawn at different times and spaces by SAS software (SAS studio https://welcome.oda.sas.com/) to directly display the development and changes of the epidemic situation and examine the epidemic law by layers. Confirmed case scatter plots, column diagrams and line diagrams were also used to display the time trend. Spatial stratified heterogeneity of incidence rate in China was analysed by R software using the package “Geodetector” [13, 14] and was described through q statistic. The city incidence rate was considered as a dependent variable and the province was considered as an explanatory variable in this study. Furthermore, the spatial autocorrelation was analysed by Arcgis software and was described through the Global Moran’s Index [15, 16] and hot spot analysis. Global Moran’s Index was proposed by the Australian statistician Patrick Alfred Pierce Moran [15] to assess the overall pattern and spatial agglomeration. The hot spot analysis could identify statistically significant spatial clusters of high values (hot spots).

## Results

### Prevalence in Sichuan Province (non-foreign imported cases)

#### Geographical distribution

**Fig. 1:**
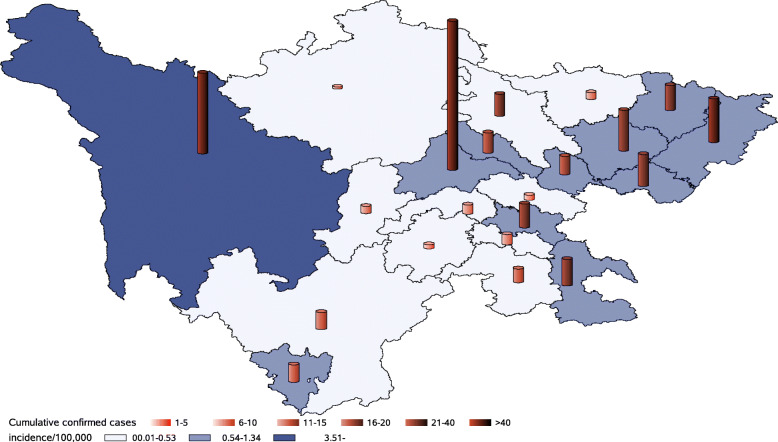
City-level distribution of mainland confirmed cases in Sichuan Province (up to March 4). *Each city of Sichuan Province (21 in total) has reported confirmed cases, with a total mainland case number of 539 and an incidence rate of 0.6462/100,000. SAS software was used to create the map (SAS studio online, https://welcome.oda.sas.com/)

**Fig. 2:**
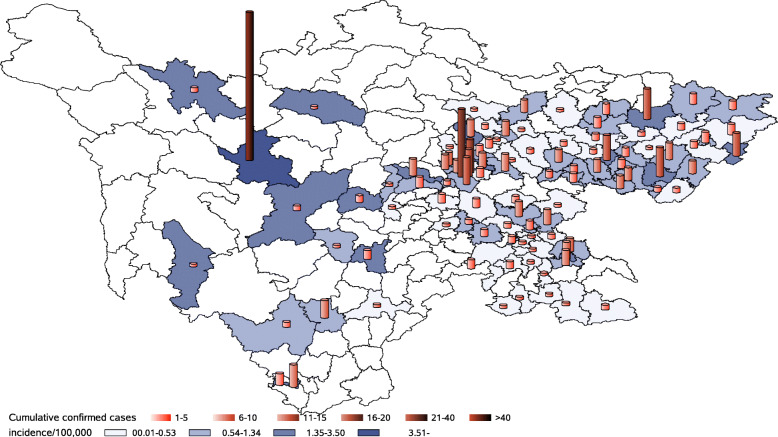
County-level distribution of mainland confirmed cases in Sichuan Province (up to March 4). *The confirmed cases were distributed in 103 counties, accounting for 56.28% of Sichuan Province. The regions with the highest number of cases and counties are located in the eastern part of Sichuan Province, which is directly adjacent to Chongqing and closer to Hubei Province. SAS software was used to create the map (SAS studio online, https://welcome.oda.sas.com/)

On March 4, Sichuan Province ended the increase in confirmed cases on the mainland (the first wave). Each city of Sichuan Province (21 in total) reported confirmed cases, with a total mainland case number of 539 and an incidence rate of 0.6462/100,000. Analysis of confirmed cases was based on their current address, with 48.33% in urban and 51.67% in rural areas. The top five cities with confirmed case numbers were Chengdu, Ganzi, Dazhou, Nanchong, and Guang’an. The cities with top five incidence rate were Ganzi, Panzhihua, Guang’an, Chengdu, and Dazhou (Table 1). Moreover, the confirmed cases were distributed in 103 counties, accounting for 56.28% of Sichuan Province (183 in total). Among them, 3 cities (out of 21 cities) found cases reported in all their counties. Geographically, the regions with the highest number of cases and counties were located in the eastern part of Sichuan Province, which is directly adjacent to Chongqing and closer to Hubei Province. Although the number of confirmed cases in the eastern counties was relatively high, a small number of counties in the western part also had a high incidence (Table 1, Figs. 1 and 2).

**Table 1:** Number and incidence of COVID-19 in different cities of Sichuan Province

City	Population	Number of counties	Confirmed case number	Rank of case number	Incidence /100,000	Rank of incidence
Cheng Du	1633	20	144	1	0^.^8818	4
Gan Zi	119^.^6	18	78	2	6^.^5217	1
Da Zhou	572	7	42	3	0^.^7343	5
Nan Chong	644	9	39	4	0^.^6056	7
Guang An	324^.^1	6	30	5	0^.^9256	3
Ba Zhong	332^.^2	5	24	6	0^.^7225	6
Lu Zhou	432^.^4	7	24	6	0^.^555	9
Nei Jiang	369^.^9	5	22	8	0^.^5948	8
Mian Yang	485^.^7	9	21	9	0^.^4324	12
De Yang	354^.^5	6	19	10	0.536	10
Sui Ning	320^.^2	5	17	11	0^.^5309	11
Pan Zhi Hua	123^.^6	5	15	12	1^.^2136	2
Liang Shan	490^.^8	17	15	13	0^.^3056	15
Yi Bin	455^.^6	10	12	14	0^.^2634	17
Zi Gong	292	6	9	15	0^.^3082	14
Mei Shan	298^.^4	6	8	16	0^.^2681	16
Ya An	154	8	6	17	0^.^3896	13
Guang Yuan	266^.^7	7	6	17	0^.^225	18
Zi Yang	251^.^2	3	4	19	0^.^1592	19
Le Shan	326^.^7	11	3	20	0^.^0918	21
A Ba	94^.^4	13	1	21	0^.^1059	20
Subtotal^a^	8341	183	539		0^.^6462	
Overseas imported cases			21			
Imported case after Wuhan unsealed			1			
Total			561			

^a^In Sichuan, up to March 4, a total of 21 cities had been reported (covering all the city-level geographical units), with a subtotal number of 539 confirmed cases and an incidence of 0.6432/100,000. The subtotal number was for the confirmed first wave. It did not contain 21 overseas imported cases reported from March 17 to April 6 and only one was imported from Wuhan after Wuhan was unsealed. Wuhan was unsealed on April 8, and this case was reported on April 17. All 22 cases were discovered in the observation period after they returned to Chengdu and did not cause secondary transmissions

#### Time distribution

**Fig. 3:**
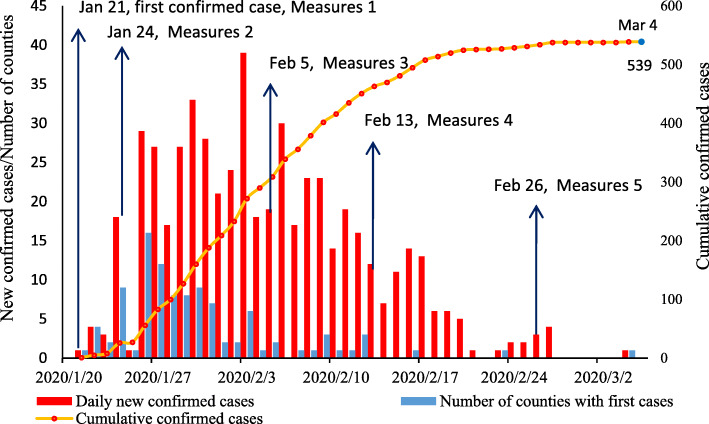
Time distribution of the number of confirmed cases and counties with the first diagnosed cases in Sichuan Province (mainland cases). *The maximum number was the third and fourth days after the Wuhan lockdown. The rapid increase was particularly obvious in the first two weeks. January 21, with the first confirmed case, the Sichuan CDC launched an emergency response. January 24, Sichuan government launched the first-level response to major public health incidents. February 5, commencement of district classification prevention and control at the city level. February 13, commencement of district classification prevention and control at the county level. February 26, Sichuan government reduced the response to level 2. March 4, the last local cases in the first wave of the epidemic. March 18, establishment of a thematic working group on overseas imported cases. March 25 reduced the response to level 3. From March 17 to April 6, a total of 21 overseas imported cases were reported in Sichuan

The time of the first confirmed case report in the 21 cities of Sichuan Province mainly came one week after the Wuhan lockdown (January 24–January 31) [17]. At this stage, the current addresses of the first confirmed cases were distributed in 70 counties, accounting for 68% of the reported counties (103 in total) in Sichuan Province on March 4. The maximum number of cases was on the third and fourth days after the Wuhan lockdown (January 26–27). During those two days, 28 counties (27%) reported their first case. The rapid increase in the confirmed number was concentrated in the four weeks after the closure of Wuhan (January 24–February 20), and the increase was particularly obvious in the first two weeks (Fig. 3).

### Prevalence in neighbouring provinces of Sichuan and other parts of China

#### Spatiotemporal distribution

On December 31, 2019, 27 cases were first reported on the official website of Hubei Province, all in Wuhan; on January 23, 2020, Hubei announced that it had entered a Grade II response and the Wuhan lockdown began [18]. Of the 835 cases reported nationwide (including 5 in Hong Kong, Macao, and Taiwan), 549 were in Hubei (495 of these in Wuhan) and 267 were in all provinces outside Hubei. A total of 15 cases in Sichuan Province were distributed in 7 cities (the first imported case was diagnosed on January 21), ranking 9th in China. The number of cases in Chongqing, adjacent in eastern Sichuan, was the only neighbouring province with more cases than Sichuan (27 cumulative cases). The epidemic situation in Yunnan and Guizhou adjacent in southern Sichuan was relatively light. Twenty-eight days later, on February 20, a total of 75,993 cases (including Hong Kong, Macao, and Taiwan, which accounted for 102 cases, the total number of which were corrected based on Hubei’s revision) were reported, of which 63,088 were in Hubei (45,346 of these were in Wuhan). The revision was originally made by the Health Commission of Hubei Province website on February 21, raising the total to 12,905 cases in provinces outside Hubei (including in Hong Kong, Macao, and Taiwan). A total of 525 cases in Sichuan Province were distributed in all 21 cities, ranking 11th in China, next to Chongqing. Fourteen days later, on March 5, a total of 80,710 cases (including 158 cases in Hong Kong, Macao, and Taiwan) were reported nationwide, including 67,592 cases in Hubei (49,797 of these in Wuhan) and a total of 13,118 cases in provinces outside Hubei (including Hong Kong, Macau, and Taiwan). A total of 539 cases in Sichuan were distributed in all 21 cities, still ranking 11th in China. The number of new cases nationwide fell below one hundred the next day.

**Fig. 4:**
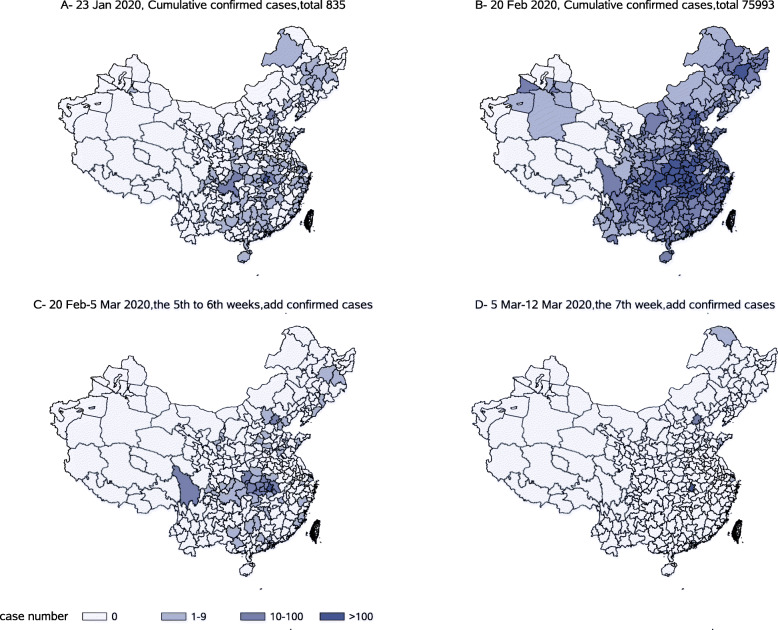
Spatiotemporal distribution of the epidemic (comparison of key dates, local cases). *Part **a** shows the case distribution in each city on January 23 (Wuhan lockdown). Part **b** shows the case distribution on February 20 (fourth week after the closure of Wuhan). Part **c** shows the increase in the number of cases in the fifth and sixth weeks after Wuhan lock down. Part **d** shows the increase in the number in the seventh week. SAS software was used to create the map (SAS studio online, https://welcome.oda.sas.com/)

The concentrated outbreak time of confirmed cases was the first 4 weeks after the implementation of the lockdown measures (Fig. 4). On December 31, cases were only reported in Wuhan. Later, on January 19, the first confirmed case was reported outside Wuhan in Shenzhen of Guangdong Province. From December 31 to January 23 (i.e., 118 cities reported cases on the day of Wuhan lockdown), the number of confirmed cases increased by a factor of 31. In the next four weeks after the Wuhan lockdown, 90% of the cities nationwide were affected, and the number of cases increased by a factor of 91, compared with January 23. In the fifth and sixth weeks, the number of newly confirmed cases and the number of reported areas slowed down significantly, with an increase of only a factor of 0.06 (63 cities except Wuhan increased with a total of 473 cases, and the average daily increase was 0.53 cases/city). In the 7th week (March 5–March 12), there was only a small increase of 62 cases in 10 cities of China except for Wuhan.

**Fig. 5:**
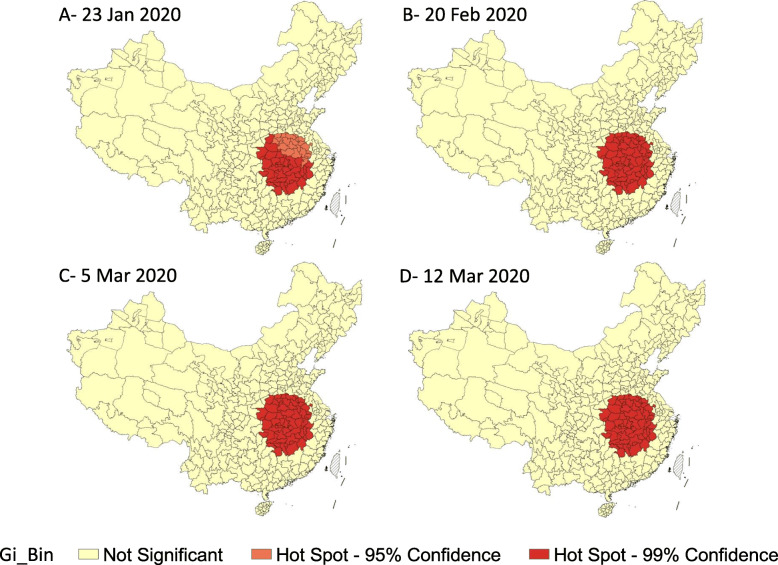
Hot spot analysis of the epidemic (comparison of key dates, local cases). *Spatial autocorrelation analysis of China showed positive spatial correlation. From January 23 to February 20, the epidemic situation was rapidly gathering in space. In part A, there were 39 level 1 hot spots,22 level 2 hot spots. The level 1 hot spots were mainly distributed in Hubei, Hunan, Jiangxi Provinces. The level 2 hot spots were mainly distributed in Henan and Anhui Provinces. In part B, all the original level 2 hot spots turned into level 1. After February 20, the hot spots did not expand, which reflected that the spread of the epidemic was effectively controlled

The q statistics values of spatial stratified heterogeneity of city incidence among provinces in China (excluding Hong Kong, Macao and Taiwan) on January 23, February 20, March 5 and March 12 were 0.16, 0.37, 0.35 and 0.35 (all the *p* value < 0.05), which revealed that the heterogeneity existed and the province as a space factor explained 16, 37, 35 and 35% of the city incidence. The Global Moran’s Index [15] of spatial autocorrelation analysis on January 23, February 20, March 5 and March 12 were 0.02, 0.20, 0.18 and 0.18 (all the p value < 0.05). All the Global Moran’s Index values were positive expressed that the epidemic in China tending to cluster spatially. The results also showed that from January 23 to February 20, the epidemic situation was rapidly gathering in space. Besides, the spread of the epidemic slowed down after February 20, and the spatial concentration decreased slightly. Hot spot analysis further demonstrated that the epidemic gathered in Hubei, Hunan, Jiangxi Provinces on February 20 and did not expand, which suggested that the spread of the epidemic was under effective control after February 20 (Fig. 5).

#### Distribution of the epidemic length in different regions

**Fig. 6:**
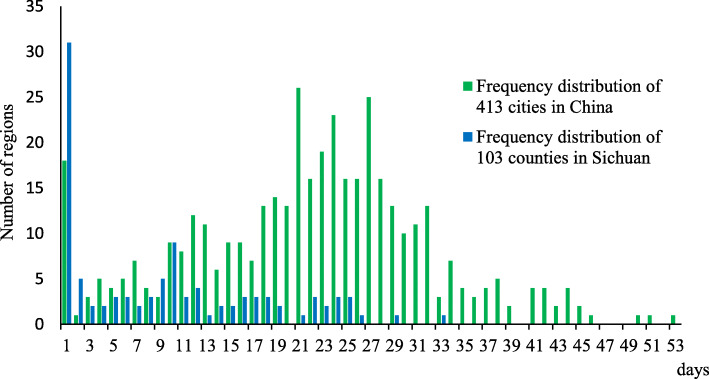
Analysis of the epidemic length (local cases) in China and Sichuan Province. *Here, zero growth excludes overseas imported cases as well as their associated cases and a very few cases who leave Wuhan after it was unsealed on April 8. Briefly, the length was calculated by the information of occurrence and the end of local cases in the first wave

**Fig. 7:**
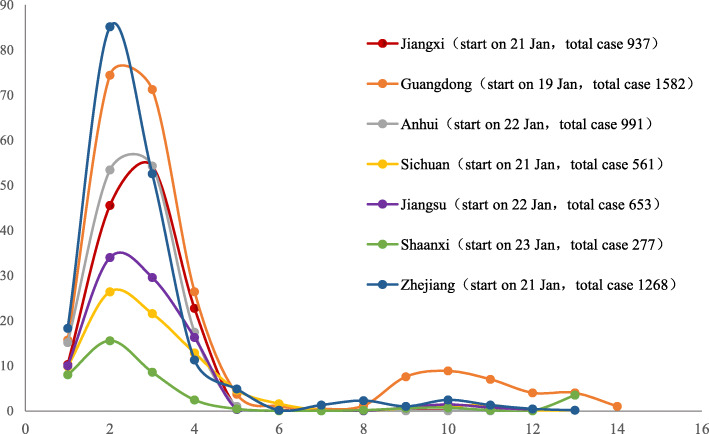
The comparison of average daily increments between Sichuan and the other 6 provinces. *One unit of the abscissa was a week (7 days). It set the day that the first cases reported in each province as the starting point. During the two weeks before the Hubei lockdown, the top destination of Hubei’s emigration population was Henan Province. Sichuan Province ranked seventh. Taking the migration from Hubei at this stage as a possible high-risk factor, this chart selected the provinces that rank close to Sichuan Province (rank 4 to 10) for comparison. The peak time in Sichuan Province was earlier (occured in the second week according to the weekly analysis, ranked the first in common), and the peak was lower (26.7 cases average daily increment in that week, only higher than Shaanxi). The fluctuation after the eighth week of each province mainly came from imported cases abroad. The data go up to April 21 in this figure

On March 12, in the seventh week after the closure of Wuhan, the growth of local cases in the first wave ended in all the cities in China (excluding Wuhan, Taiwan and Hong Kong). Based on statistics at the city level (the districts of municipalities were analysed as a city unit according to the high population density of municipalities), the length calculated by the date of occurrence and the end of local cases in a total of 413 cities were used for analysis. The study shows that the longest date length from the first case confirmed to zero growth in each city was 53 days, and the average and median lengths were 22 and 23 days, respectively. The 1st quartile and 3rd quartile were 15 and 28 days, respectively. When the length was 21 days, the frequency of the cities was highest at approximately 26 cities (6.3% in 413 cities). Further analysis of the epidemic length at the county level (regional unit) in Sichuan Province included a total of 103 counties that had epidemics. The results showed that there was no local case increase after March 4. The largest date length from the first confirmed case to zero growth was 33 days, with a mean and median of 9 days. The 1st quartile and 3rd quartile were 1 and 16 days, respectively. The highest city frequency of the date length was 1 day for approximately 31 cities (30% in 103 cities) (Fig. 6). Taking the migration from Hubei during the two weeks before its lockdown as a possible high-risk factor, this study compared the provinces for which the migration rank was close to Sichuan Province and found that the peak time in Sichuan Province was earlier (occured in the second week according to the weekly analysis, ranked the first in common) and the peak value was lower (26.7 cases average daily increment in that week, only higher than Shaanxi) (Fig. 7). According to the daily analysis, the peak of Sichuan appeared only one day later than Zhejiang among the 7 provinces, but its peak value was much lower than Zhejiang Province (35 cases compared to 132 cases).

### Global situation during the same period

**Fig. 8:**
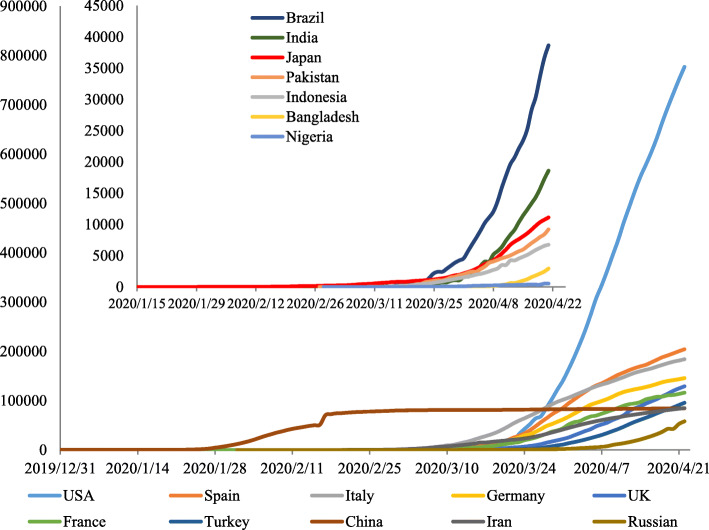
Epidemic trends in major global countries. *The length of time from the first report to the period of rapid rise in different countries varied based on the difference of applicable conditions of nucleic acid detection and the national control strategies. The ordinate represents the cumulative number of confirmed cases. The lower right curves indicate the countries with the top 10 confirmed cases worldwide on April 21. Most of them entered the rapid growth phase within 1 month. The top left curves indicate the countries with the top 10 populations (except the USA, China, and Russia, which are already in the lower right). Most of them were starting to show a strong upward trend, which suggested a high risk in the next few weeks

The earliest confirmed cases outside China were reported in Thailand on January 13, followed by Japan (January 15), the Republic of Korea (January 20), the USA (January 21), Singapore and Vietnam (January 23). On February 21, four weeks after the first reported case in Korea, the number of confirmed cases began to rise rapidly. In addition, cases in Italy began to rise rapidly two weeks after the first case was reported there, and cases in Iran increased quickly in the second week after the first case report. Furthermore, the USA (5 weeks after the first case) and other European countries such as Spain (3 weeks after the first case), France (5 weeks after the first case), Germany (4 weeks after the first case), and Switzerland (1 week after the first case) began to see a rapid rise in infections. Overall, many European countries experienced that rise earlier than the USA. The length of time from the first case reported to the period of rapid rise in different countries varied. Most of them entered the rapid growth phase within 1 month (Fig. 8). The confirmed cases reported in the USA increased sharply in weeks 8 to 9.

**Fig. 9:**
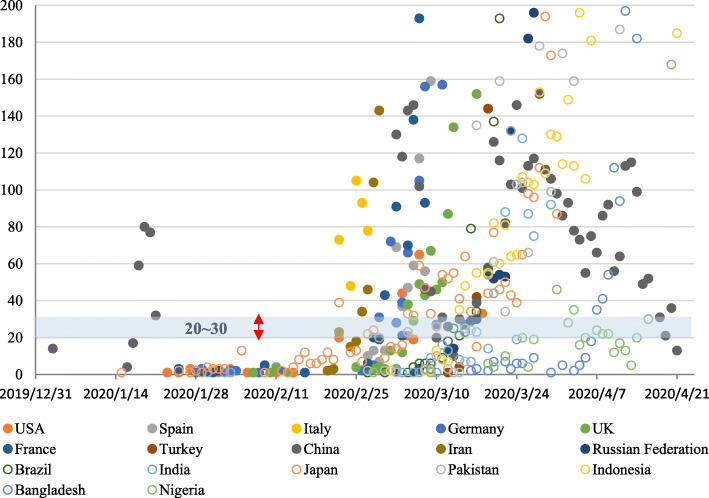
A scatter plot of confirmed cases. *The ordinate represents the daily growth number of confirmed cases. The solid circles indicate the countries with the top 10 confirmed cases. The hollow circles indicate the countries with the top 10 populations. The solid circles suggest that when the daily increase exceeded 20–30 cases, most countries entered an obvious increase stage

**Fig. 10:**
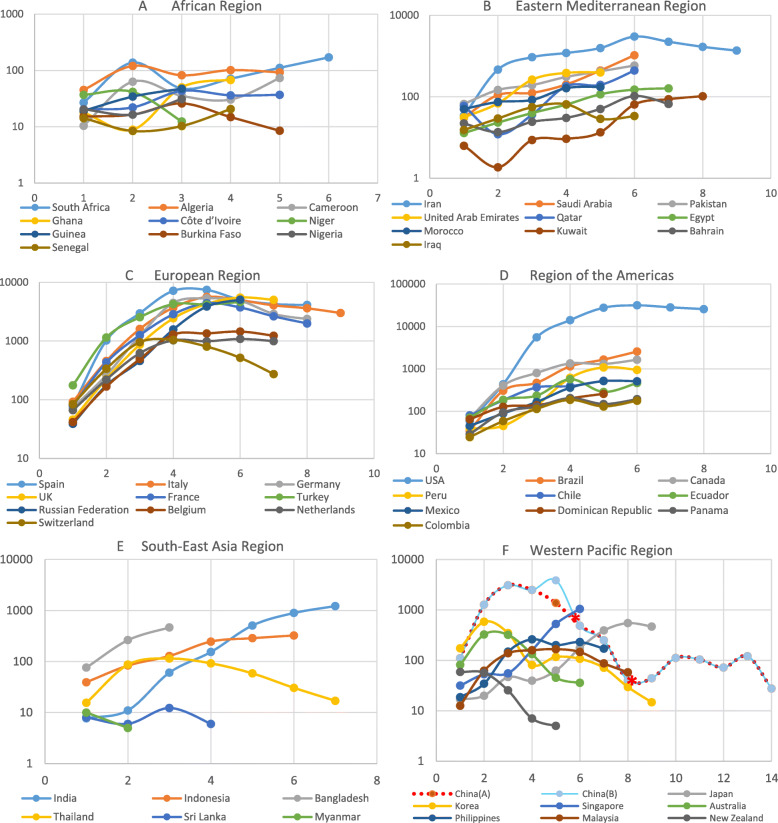
The analysis of the average daily increment, per 7 days, in different countries and regions. *One unit of the abscissa was a week (7 days). It set the day when the new cases exceeded 20 as the starting point. According to the regional division in the WHO report, this figure included the top 6–10 countries of each region, for which the daily increase exceeded 20, to show the trends of confirmed cases. Countries meeting this condition in the Southeast Asia region and Western Pacific region were just 6 and 8, respectively, both less than 10. The countries in parts **a** and **e** were closer to the equator with slow growth or at the early stage of infections. In parts **b** and **d**, most countries were in the rapid growth phase, as well as Russian in part **c**. Dividing the average daily increment value of the final week by the previous week, to get the change coefficient for each country here. The average change coefficient value of the countries in a region was used to evaluate the growing speed of a region. The average change coefficient was 1.3 in the Eastern Mediterranean region and 1.25 in the region of the Americas. Besides, the change coefficient was 1.3 in Russian. Most European countries in part **c** as well as the USA in part **d** presented an inflection point or at a plateau period in the fourth to fifth weeks but declined slowly. Germany, Spain, Italy, and China (**a**) took 28, 29, 34, and 18 days, respectively, to reach their peak daily increments. China (**b**) took 27 days as it contained an increase in clinical diagnoses published in the fifth week. The curve of China dropped rapidly after the fifth week. The two stars in the curve of China indicate the start of reporting imported cases from abroad (starting on February 26) in mainland China (excluding Hong Kong, Macao and Taiwan) and the response of suspending entry of foreigners holding valid Chinese visas and residence permits (March 28). From February 26 to April 21, mainland China reported approximately 1610 imported cases from abroad, approximately 3 times the Sichuan cumulative cases. During that time, there were 174 newly confirmed local cases in mainland China. All the newly confirmed case numbers were in the period up to April 21 in this figure

**Fig. 11:**
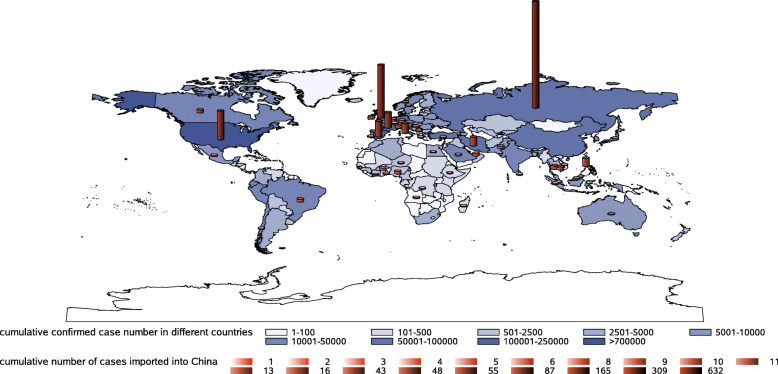
Global distribution of confirmed cases and source distribution of cases imported into China from abroad. *With basically the disappearance of local cases in China and the rapid rise of epidemics in other countries abroad, a large number of cases abroad have been re-imported into China. The overseas imported cases were first reported in Ningxia Province in mainland China (excluding Hong Kong, Macao and Taiwan) on February 26 and totalled 1610, imported from 48 overseas countries (1601 cases were collected their information of input countries), on April 21 and distributed in 19 provinces in China. SAS software was used to create the map (SAS studio online, https://welcome.oda.sas.com/)

From the scatter plot of confirmed cases (top 10 countries), it was found that when the daily increase exceeded 20–30 cases, most countries entered an obvious increase stage (Fig. 9). From the time point of the first daily increase of 20 cases, Germany, Spain, and Italy reached the peak of daily incremental rising after 28, 29, and 34 days, respectively, and then the daily increment showed a downward trend. The average daily increment of a 7-day rolling timeframe also showed an inflection point in Italy. The countries of France, the UK, and the USA appeared to peak at 36 days, 32 days, and 44 days, respectively. According to the regional division in the WHO report, many countries in the African region and Southeast Asia region were closer to the equator with slow growth or only at the early stage of infection spread. This study also analysed the top 10 countries of confirmed cases in each region (Fig. 10, data up to April 21). Firstly, dividing the average daily increment value of the final week by the previous week, to get the change coefficient for each country here. The change coefficient was used to express the growing speed of a country. The average change coefficient value of the countries in a region was used to evaluate the growing speed of a region. The average change coefficient was 1.3 in the Eastern Mediterranean region and was 1.25 in the region of the Americas. Besides, the change coefficient was 1.3 in Russian. Secondly, most European countries presented an inflection point or were at a plateau period in the fourth to fifth weeks but were falling slowly. The curve of cases in China dropped rapidly after the fifth week. On February 13–15, Hubei Province in China reported the clinical diagnosis. The number of clinical diagnosis cases was counted in the WHO COVID-19 Situation Report-28 with a statement that this was only applicable to Hubei Province. If the clinical diagnosis cases were not taken into account, China reached the peak of the daily increment of the epidemic in 18 days, and if such cases were taken into account, China peaked in 27 days. China has been subjected to imported cases since the eighth week, with basically a disappearance of local cases and the rapid rise of epidemics in other countries abroad (Fig. 11). The overseas imported cases were first reported in Ningxia Province in China on February 26 and cumulatively included 1610 as of April 21, 21 of which were reported by Sichuan Province.

## Discussion

The eastern region of Sichuan Province is adjacent to Chongqing and Hubei provinces. Many counties there have reported cases, which is related to the fact that imported cases were the main cases in Sichuan Province in the early stage. Combined with the “Baidu Migration Data” analysis [19], the national migration scale index [19] on January 23 and earlier was slightly higher than that of the same period last year (January 24 was the Chinese New Year’s Eve), but after January 25, the national migration scale index fell slightly until March 12, which was lower than the same time of last year. On January 23, for the proportion of migrants in cities across China (the ratio of the population that moved to a city to the total population that moved into different kinds of cities in whole country), the top 3 were Chongqin, Chengdu, and Zhoukou city. Twelve of the top 100 cities were in Sichuan Province and all of these are located in the eastern part. Among them, Chengdu ranked first in the 26–30-day timeframe. The proportion of emigration place (the ratio of the emigration to a certain city to the total emigration for the whole country), 9 of the top 100 were in Sichuan Province, and were also located in the east. This result suggested that the population flow in eastern Sichuan was relatively large. In addition, the overall low incidence in western Sichuan was also associated with a lower population density in the western region than in the east. In a few of these areas, the incidence was high because a few people, who went back from Chengdu in the incubation period, participated many aggregation activities there [20].

The first reports of the kinds of cities in Sichuan Province were concentrated in the first week after Wuhan instituted a lockdown. This reflected the rapid response and quarantining of Sichuan Province. The number of cases increased significantly in the first 2 weeks, which was in line with the usual incubation period [21, 22]. This suggested that the first two weeks after the occurrence of the first case was a critical stage for establishing quarantine, prevention and control measures.

The spatiotemporal distribution of the country suggests that the virus spreads rapidly during the two incubation periods. The analysis of the length of the epidemic indicates that after China adopted strong prevention and control measures, most regions can control the growth rate of the epidemic in approximately 3 weeks. The average and median of the epidemic length of county-level regional units are significantly lower than the epidemic length of city-state regional units. It suggested that regional units of different levels can combine actual conditions to formulate prevention and control measures, duration of prevention and control, and resumption time. Especially for areas where there is no community outbreak in the local area, production can be resumed early to reduce economic losses. The increased number of COVID-19 case in spatiotemporal distribution showed that the epidemic in China went through mainly two stages of release. In the first stage, the 3 weeks since the Wuhan reported, the confirmed case increased and the epidemic spread rapidly (Part A of Fig. 4). In the second stage, the next four weeks after the Wuhan lockdown, the release was accelerated and was then quickly close to the end of the first epidemic wave (Part B, C and D of Fig. 4). Results indicated that on March 12, the case incidence in major countries worldwide was still in the first stage, and from March 25 onward, most countries entered the second stage of rising cases. After comparing and analysing the morbidity, we found that the length of time from the first case to the rapid rise of cases varies in different countries. Most countries entered the period of rapid growth after 4 weeks. The delayed entry of rapid growth in a few countries was related to the late launch of extensive testing.

China has taken many effective measures to address the epidemic. Very important activities included well-coordinated prevention, control and treatment and reflected 1) centralized and efficient command, 2) a tight prevention and control system involving all sectors of society, 3) an all-out effort to treat patients and save lives, 4) information that was released in an open and transparent manner, and 5) science and technology underpinnings. In addition, the government assembled a powerful effort to beat the virus. This fight mobilized the whole country with a message that lives are precious, which let the people unite as one. During the battle against the virus, the government did much to coordinate prevention and control with social and economic development. Moreover, the whole process of fighting the epidemic could not be separated from the support of the international community and Chinese citizens overseas.

COVID-19 is one of the most significant public health emergencies that has occurred in China since the founding of the People’s Republic of China. In the early stage of the epidemic, due to insufficient resources for medical treatment and the failure to connect quickly with the disease prevention and control information system in a few newly designated hospitals, as well as the overloaded operations in hospitals and the very busy treatment schedule for medical staff late reports, missed reports and false reports occurred. On March 18, the newly confirmed growth was reported to be zero in Wuhan for the first time. Since then, the spread of the epidemic has been basically blocked, and the control of the travel from Wuhan to other cities has been lifted, creating favourable conditions for the comprehensive and detailed verification and revision of the epidemic data. The revised types included reductions and additions. Among them, 217 cases were removed after verification (some patients had been treated in more than one district or in multiple hospitals, resulting in duplicate reporting), and 542 cases were added (previous cases not reported in time due to late reporting and omission). The total number of confirmed cases was revised from 50,008 to 50,333 on April 17 based on the principles of objectivity and the transparency of data [23]. This part of the newly confirmed number accounted for a small proportion (0.6%) of the total confirmed number in Wuhan. The limitation of this study was that the 0.6% confirmed number was presented centrally on April 17 in Figs. 8, 9 and 10, because the data released by Health Commission of Hubei Province were not assigned to the date of previous diagnosis.

## Conclusions

Considering the history of the epidemic in China, it is suggested that even if countries take effective and powerful measures, the number of confirmed cases will increase rapidly in the first month, which will inevitably challenge the medical resources and population health of those countries. If no measures are taken, it is difficult to estimate the number of infections and the overall economic losses that would be caused. Combined with the severity of the illness and mortality rates, the health costs of the population would be substantial.

In addition, upon entering the period of zero growth and the gradual cure of local cases in China, high vigilance must be maintained. From February 26, 2020, the different provinces of China began to report imported cases from abroad. With the change in the epidemic situation in different countries and the different measures taken by governments, the cases imported into China have soared. A small number of overseas associated cases have appeared. In response, from March 28, 2020, the entry of foreigners holding valid Chinese visas and residence permits were suspended. Some countries with improved epidemic situations also need to quickly formulate a continuous “local strategy of entry checkpoints” to fend off imported COVID-19 in the context of the global epidemic. Until vaccines are widely available, these governments need to be highly vigilant about localized outbreaks.

## Data Availability

The city-level COVID-19 confirmed case number information for Sichuan was made available from the Health Commission of Sichuan Province website [20]. The county level information of Sichuan was obtained by applying it to the Surveillance System of Sichuan CDC. The confirmed case number information of other provinces was available from the National and province level Health Commission website [5, 6]. Data from various countries were from the WHO COVID-19 Daily Report published since January 21,^7,^ which also contained the confirmed information of the different provinces in China before the date of March 15. The case number information before the first WHO reports came from different national government websites [8–11]. The confirmed number data in the WHO reports could also be downloaded from the GitHub website, collated as a public database continuously updated by the Johns Hopkins University Center for Systems Science and Engineering [24]. The population of Sichuan Province was available from the Statistical Yearbook of Sichuan Province [12].

## Abbreviations

COVID-19: Coronavirus disease
CDC: Centers for Disease Control and Prevention
Korea: Republic of Korea
MERS-CoV: Middle East Respiratory Syndrome Coronavirus
SARS-CoV: Severe acute respiratory syndrome coronavirus
SARS-CoV-2: severe acute respiratory syndrome corona virus 2
UK: the United Kingdom
USA: the United States of America
WHO: World Health Organization
2019-nCoV: 2019 novel coronavirus, tentatively named SARS-CoV-2
